# Factors Influencing the Multicultural Competence of Occupational Therapists in South Korea

**DOI:** 10.1155/oti/3677844

**Published:** 2026-07-11

**Authors:** Oan Na Lee, Gyeong-A Park

**Affiliations:** ^1^ Psychological Counselor, Samsung Heavy Industries, Geoje, Republic of Korea, samsungshi.com; ^2^ Department of Occupational Therapy, Chosun University, Gwangju, Republic of Korea, chosun.ac.kr

**Keywords:** multicultural competence, multicultural empathy, occupational therapists, personality trait, self-esteem

## Abstract

**Purpose:**

Multicultural competence is an essential skill for healthcare professionals, particularly in multicultural contexts. It is important to examine the factors that influence multicultural competence among health professionals. This study aimed to investigate the effect of various factors on multicultural competence of occupational therapists in South Korea.

**Methods:**

A total of 153 occupational therapists (aged 23–55 years) in South Korea completed a set of self‐report questionnaires, including the Scale of Ethnocultural Empathy (SEE), Big Five Inventory (BFI), Rosenberg Self‐Esteem scale (RSES), and Cultural Competence Assessment Inventory (CCAI). Data were analyzed using IBM SPSS Statics Version 27.0. To examine the relationships among demographic variables, multicultural empathy, personality traits, self‐esteem, and multicultural competence, descriptive statistics, Pearson correlation analysis, and multiple regression analysis were conducted.

**Results:**

Openness, multicultural empathy, self‐esteem, and English proficiency were significant positive predictors of multicultural competence, explaining 52.8% of its variance. These findings indicate that personal and experiential factors substantially contribute to enhancing multicultural competence among occupational therapists.

**Conclusion:**

Training programs aimed at improving multicultural competence should incorporate approaches that enhance occupational therapists’ English proficiency, multicultural empathy, openness, and self‐esteem. Strengthening these personal and experiential attributes can promote higher levels of multicultural competence and enable occupational therapists to deliver culturally sensitive and effective care in diverse clinical settings.

## 1. Introduction

As globalization accelerates, cultural diversity within nations has become a defining feature of modern societies. South Korea is no exception, as it continues to evolve into a multicultural nation. According to the Ministry of Justice (2024), the number of individuals from diverse cultural backgrounds—including marriage migrants and their children, international students, and migrant workers—has exceeded 2.6 million, representing approximately 5.2% of the total population [1]. As people from various cultural backgrounds increasingly coexist, opportunities for intercultural interaction have expanded. Consequently, promoting mutual understanding, harmony, and effective communication among culturally diverse groups has emerged as a key challenge in contemporary Korean society [2].

Healthcare professionals are among those who most frequently interact with individuals from diverse cultural and linguistic backgrounds. Following the revision of the Medical Service Act in 2009, the attraction of foreign patients became legally sanctioned, leading to a continuous increase in the number of international patients seeking medical services in Korea. According to the Korea Health Industry Development Institute (2024), the cumulative number of foreign patients who have received medical treatment in Korea has surpassed 5 million [3].

As the multicultural population in Korea continues to grow—including marriage immigrants and their families, international students, migrant workers, and foreign patients—healthcare professionals face increasing demands to communicate effectively with patients from various cultural backgrounds and to ensure optimal treatment outcomes. In particular, occupational therapists, who play an essential role in the rehabilitation process, must develop a high level of cultural competence to engage effectively with patients from different cultures and to enhance the quality and efficacy of rehabilitation interventions [4].

Occupational therapists are healthcare practitioners who work closely with individuals representing diverse cultural perspectives. To perform their professional roles effectively, they must possess well‐developed interpersonal and communication skills [4]. Because patients may hold values, beliefs, and behaviors that differ from those of their therapists, the ability to understand and interact across cultural boundaries is crucial. Therefore, healthcare professionals, including occupational therapists, are required to cultivate cultural competence as a core professional competency [5].

Cultural competence refers to the ability of health care providers to understand, respect, and respond appropriately to clients from different cultural contexts [6]. According to Campinha‐Bacote, cultural competence consists of five interrelated constructs: cultural awareness, cultural knowledge, cultural skill, cultural encounters, and cultural desire. Together, these constructs represent the essential dimensions that enable healthcare providers to deliver effective and culturally responsive care to diverse populations. The multicultural competence of occupational therapists, as healthcare service providers, is reflected in their ability to enhance cultural awareness through self‐reflection, accumulate cultural knowledge, develop proficiency in relevant knowledge and skills, and effectively engage with diverse cultural encounters [6].

As emphasized by the American Journal of Occupational Therapy, multicultural competence is a fundamental competency required of occupational therapists [4]. Occupational therapists must develop multicultural competence and acquire cultural competency to effectively provide therapeutic interventions for clients from diverse cultural backgrounds [4]. Therefore, it is important to identify the personal factors that influence the multicultural competence of occupational therapists.

In a study exploring strategies to enhance cultural competence among occupational therapists in the United States, individual factors such as critical thinking, self‐awareness, problem‐solving skills, and an understanding of cultural considerations were emphasized as essential components for strengthening cultural competence [5]. These personal factors contribute to enhancing the components of multicultural competence by reducing differences arising from cultural diversity and promoting effective intercultural communication.

Previous studies have shown that various personal factors may influence an individual’s multicultural competence. One such factor is self‐esteem, which refers to the overall evaluation of oneself [7]. Self‐esteem has been found to affect multicultural competence. Specifically, Song and Yang reported that nursing students with higher self‐esteem demonstrated greater levels of multicultural competence [8]. Positive self‐evaluation and self‐esteem may contribute to the enhancement of multicultural competence by promoting active engagement and motivation toward culturally diverse experiences [9].

In addition to self‐esteem, ethnocultural empathy has also been identified as a significant factor influencing multicultural competence. An empathetic attitude characterized by understanding, caring for, and accepting diversity has been shown to enhance the multicultural competence of health care providers [10]. Ethnocultural empathy is a fundamental component of cultural competence, reflecting a psychological disposition to understand, appreciate, and accept cultural diversity [11]. Education on culture and positive attitude toward cultural competence were identified as significant predictors of the factors influencing occupational therapists’ cultural competence [12]. It has been suggested that cultural awareness and knowledge, cultural skills, and institutional support are necessary to enhance cultural competence; moreover, cultural awareness and empathy have been identified as key factors in its improvement [12].

Furthermore, multicultural competence is conceptualized as a multidimensional construct encompassing cognition, behavior, and personality domains [13]. Reynolds and Rivera suggested that an individual’s personality traits may also influence multicultural competence. An individual’s cognitive and behavioral characteristics are expressed through attitudes the surrounding environment and situational contexts, whereas personality traits contribute to information processing, adapting, and communicating within multicultural settings [14]. Therefore, when an individual’s ethnocultural empathy and personality traits are combined, multicultural competency is expected to be further improved. However, there remains a lack of empirical research examining how personality characteristics affect multicultural competence.

An individual’s multicultural competence can be developed and enhanced through foreign language proficiency, interactions with colleagues from diverse cultural background, and varied cultural experience. A study examining the relationship between multicultural competence and demographic characteristics among nurses reported significant associations with gender, level of education, proficiency in foreign language, variables related to multicultural exposure, and bilingualism [15]. Furthermore, foreign language proficiency and cultural competence are critical factors in occupational therapy practice, and it has been suggested that occupational therapy students’ cultural awareness, sensitivity, and responsiveness to language differences positively influence their level of cultural competence [16]. Therefore, multicultural competence may be conceptualized as a composite of an individual’s general characteristics, multicultural experiences, personality traits, psychological attributes, and attitudes toward multiculturalism.

A culturally competent healthcare environment can enhance clients’ health outcomes and increase their overall satisfaction with care [17, 18]. Despite the importance of multicultural competence in healthcare, research on this topic remains limited. In particular, while numerous studies have explored the multicultural competency of nurses and health professionals in the field of psychology, there is a relative lack of research on occupational therapists. Although several studies within the field of occupational therapy have focused on occupational therapy students, there remains a paucity of research investigating the factors that influence occupational therapists’ multicultural competence, despite its recognition as a critical skill for effective professional practice. Therefore, this study aims to examine how occupational therapists’ self‐esteem, multicultural empathy attitude, and personality traits influence their multicultural competence.

The objectives of this research are as follows:

1. To investigate the level of cultural competence of occupational therapists
2. To investigate the effects of general characteristics, personality traits, cultural empathy, and self‐esteem on occupational therapists’ cultural competence

This research aims to provide effective treatment by occupational therapists through program support for enhancing multicultural competence in clinical settings.

## 2. Materials and Methods

### 2.1. Procedures

This study employed a cross‐sectional research design to examine the relationships among personality, ethnocultural empathy, self‐esteem, and cultural competence. Data were collected using a snowball sampling method. The participants were occupational therapists working in medical institutions, welfare centers, development centers, and dementia care centers across South Korea.

Data collection was conducted through an online survey administered between May 2021 and April 2023. The study protocol was approved by the Institutional Review Board of Dongshin University (IRB No. 1040708‐202001‐SB‐002). All participants were informed about the purpose of the study prior to participation. Those who consented to participate received a survey package that included a written informed consent form, a demographic questionnaire, the Korean version of the Big Five Inventory (BFI‐K), the Scale of Ethnocultural Empathy (SEE), the Rosenberg Self‐Esteem Scale (RSES), and the Cultural Competence Assessment Inventory (CCAI). A demographic questionnaire included clinical experience, experience treating patients from different culture, experience working with colleagues from different culture, and participants’ self‐assessed English proficiency levels (low, intermediate, advanced). Data on participant’s English proficiency were collected using a self‐report scale commonly employed in survey research. As these variables were considered factors influencing the participants’ cultural competence, they were included in the questionnaire by the researchers.

Participants completed the questionnaire independently by reading each item and recording their responses. After excluding seven questionnaires with incomplete or insincere responses, a total of 153 valid questionnaires were included in the final analysis [19].

### 2.2. Participants

The sample size was calculated using the G∗ power 3.1 program. With an effect size of 0.15, a significance level of 0.05, a statistical power of.95, and seven independent variables, the minimum required sample size was determined to be 153 participants [20]. A total of 160 participants were recruited through distributed flyers: however, seven responses were excluded, resulting in a final sample of 153 participants.

The demographic characteristics of the participants are presented in Table 1. The participants ranged in age from 23 to 55 years (M = 27.83, SD = 4.9), females constituted a greater proportion of the sample (*n* = 121, 79.1%) than males (*n* = 32, 20.9%). Clinical experience was 60 months or less for 113 participants (73.9%). In total, 54 therapists (35.3%) had experience working with patients from different cultures, 14 (9.2%) had experience working with colleagues from different cultures. In this study, only one participant selected the “advanced” category; accordingly, English proficiency was dichotomized into low and intermediate or advanced levels. A total of 52 participants (34.0%) were classified as having intermediate or advanced English proficiency.

**Table 1:** Demographic data (*N* = 153).

Characteristics		Frequency (*N*)	Percentage (%)
Gender	Male	32	20.9
	Female	121	79.1
Age	29≥	119	77.8
	30≤	34	22.2
Clinical experience (months)	60≥	113	73.9
	61≤	40	26.1
Experience with patients from different culture	Yes	54	35.3
	No	99	64.7
Working with colleagues from different culture	Yes	14	9.2
	No	139	90.8
English proficiency	Intermediate‐or‐advanced	52	34.0
	Lowness	101	66.0

### 2.3. Measures

#### 2.3.1. Personality Traits

Individual personality traits were assessed using the Korean version of the Big Five Inventory (K‐BFI), originally developed by John and Stivastava and later validated by Korean by Kim et al. [21, 22]. The K‐BFI comprises 15 items in total: three items each for neuroticism, extraversion, openness, conscientiousness, and agreeableness. Responses are rated on a 5‐point Likert scale ranging from 1 (*strongly disagree*) to 5 (*strongly agree*), with higher score indicating stronger expressions of each respective trait. The Cronbach’s *α* values reported by Kim et al. ranged from 0.67 to 0.82, while the Cronbach’s *α* in the present study was 0.83.

#### 2.3.2. Ethnocultural Empathy Attitude

Ethnocultural empathy was measured using the Scale of Ethnocultural Empathy (SEE) developed by Wang et al. [23]. The SEE consists of 31 items in total, including 15 items on empathic feeling and expression, seven items on empathic perspective taking, five items on acceptance of cultural difference, and four items on empathic awareness. Responses are provided on a 5‐point Likert scale ranging from 1 (*strongly disagree*) to 5 (*strongly agree*). In this study, negative‐worded items were reversed‐scored prior to analysis. Higher scores indicate greater empathy toward individuals from other cultures or heterogeneous groups. The Cronbach’s *α* reported by Wang et al. was 0.91 and the Cronbach’s *α* in the present study was 0.88.

#### 2.3.3. Self‐Esteem

Self‐esteem was assessed using the Rosenberg Self‐Esteem Scale (RSES) developed by Rosenberg and translated into Korean by Won and Lee [24, 25]. The RSES evaluates individuals’ self‐worth and attitude toward themselves. It consists of 10 items rated on a 4‐point Likert scale ranging from 1 (*strongly disagree*) to 4 (*strongly agree*), with five items assessing positive self‐esteem and five items assessing negative self‐esteem. In this study, negatively worded items were reversed‐scored prior to analysis, and higher total scores indicate high levels of self‐esteem. The Cronbach’s *α* reported by Rosenberg was 0.85 and the Cronbach’s *α* in the present study was 0.86.

#### 2.3.4. Cultural Competence

Multicultural competence was measured using the Cultural Competence Assessment Instrument (CCAI) developed by Suarez‐Balcazar [26]. This instrument was originally developed in a different culture context and subsequently underwent a translation and cross‐cultural adaptation process for use in the Korean context. The translation procedure was conducted in accordance with the cross‐cultural adaptation guidelines [27]. First, two bilingual researchers independently performed forward translation of the original instrument into Korean. A consensus version was then developed through expert panel discussions. Subsequently, a bilingual expert who was unfamiliar with the original instrument conducted a back‐translation, followed by a comparative analysis between the back‐translated and original versions. Thereafter, the author and the two bilingual researchers reviewed, revised, and refined the items to ensure their semantic and cultural appropriateness within the Korean context. Finally, a pilot survey was conducted to verify the comprehensibility and clarity of the items prior to their use in the present study. The CCAI consists of 24 items on a 4‐point Likert scale ranging from 1 (*strongly disagree*) to 4 (*strongly agree*). It assesses individuals’ ability to understand and communicate effectively with people from diverse cultural backgrounds. The instrument comprises three subscales: cultural awareness and knowledge (8 items), organizational support for multicultural practice (8 items), and cultural skills (8 items). In this study, negative worded items were reverse‐scored prior to analysis, and higher scores indicate higher levels of cultural competence. The Cronbach’s *α* in reported Suarez‐Balcazar et al. was 0.90 and the Cronbach’s *α* in the present study was 0.82.

### 2.4. Data Analysis

Data collected in this study were analyzed using SPSS Statistics Version 27.0 [28]. Statistical significance was set at *p* < 0.05. Frequency distributions, descriptive statistics, and percentages were computed for demographic variables. Means, standard deviations, reliability coefficients (i.e., Cronbach’s *α*) and normality tests were conducted for all major study variables. Pearson’s correlation coefficients were used to examine the relationships among the main variables, and multiple regression analysis was performed to identify the effects of personality traits, ethnocultural empathy, and multicultural attitudes on cultural competence.

## 3. Results

### 3.1. Cultural Competence by General Characteristics

The descriptive statistics of variables were analyzed to examine the means and standard deviations of variables (Table 2). The results indicated that cultural competence differed significantly by age (*t* = −2.163, *p* = 0.032), and English proficiency (*t* = 2.972, *p* = 0.003). Specifically, participants aged 30 years or older (M = 66.71, SD = 6.43) those with intermediate‐or‐advanced English proficiency (M = 66.73, SD = 8.22) demonstrated significantly higher levels of cultural competency. No statistically significant differences were observed based on clinical experience, experience working with patients from different cultures, or collaboration with colleagues from different cultures.

**Table 2:** Mean and standard deviation of cultural competence (*N* = 153).

Characteristics		*M* ± *S* *D*	*t*/*F (p)*
Gender	Male	66.59 ± 8.22	1.980 (0.050)
	Female	63.66 ± 7.24	
Age	29≥	63.58 ± 7.69	−2.163 (0.032)
	30≤	66.71 ± 6.43	
Clinical experience (months)	60≥	63.70 ± 7.62	−1.598 (0.112)
	61≤	65.90 ± 7.09	
Experience with patients from different culture	Yes	65.63 ± 6.97	1.655 (0.100)
	No	63.54 ± 7.74	
Working with colleagues from different culture	Yes	63.29 ± 10.34	−0.384 (0.706)
	No	64.37 ± 7.2	
English proficiency	Intermediate or advanced	66.73 ± 8.22	2.972 (0.003)
	Lowness	63.01 ± 6.84	

Abbreviations: M, mean; SD, standard deviation.

### 3.2. Descriptive Statistics of Variables

Descriptive statistics for the study variables are presented in Table 3. The mean score for personality traits—neuroticism, extraversion, openness, conscientiousness, and agreeableness—range from 7.97 ± 1.52 to 10.86 ± 1.67. Openness showed the highest mean score. For the other principal variables, the mean score for ethnocultural empathy was 109.57 ± 12.10, the mean self‐esteem score was 29.81 ± 4.47, and the mean cultural competence score was 64.27 ± 7.52.

**Table 3:** Descriptive statistics of study variables (*N* = 153).

Variables	*M* ± *S* *D*	Median	Range
Neuroticism	7.97 ± 1.52	8.0	4‐11
Extraversion	10.05 ± 1.97	10.0	3–14
Openness	10.86 ± 1.67	11.0	7–15
Conscientiousness	10.05 ± 1.90	10.0	5–15
Agreeableness	10.42 ± 1.85	11.0	6–15
Ethnocultural Empathy	109.57 ± 12.10	111.0	71–140
Self‐Esteem	29.81 ± 4.47	30.0	17–39
Cultural Competence	64.27 ± 7.52	64.0	48–81

Abbreviations: M, mean; SD, standard deviation.

Overall, the participants demonstrated moderate levels of the Big Five personality traits and relatively high levels of ethnocultural empathy, self‐esteem, and cultural competence.

### 3.3. Correlations Among Study Variables

Table 4 shows that cultural competence was significantly positively correlated with extraversion, openness, conscientiousness, agreeableness, self‐esteem, and ethnocultural empathy (*r* = 0.310–621, *p* < 0.001), whereas it was negatively correlated with neuroticism (*r* = −0.220, *p* < 0.01). Among these variables, ethnocultural empathy demonstrated the strongest positive correlation with cultural competence. These findings indicate that ethnocultural empathy was strongly associated with cultural competence. In addition, occupational therapists with higher levels of positive personality traits, self‐esteem, and ethnocultural empathy tended to exhibited greater cultural competence, whereas those with higher level of neuroticism exhibited lower cultural competence.

**Table 4:** Correlation among study variables (*N* = 153).

Variable	1	2	3	4	5	6	7	8	Multicollinearity statistics	
									Tolerance	VIF
1. Neuroticism	1								0.663	1.509
2. Extraversion	−0.390 ^∗∗∗^	1							0.543	1.842
3. Openness	−0.380 ^∗∗∗^	0.489 ^∗∗∗^	1						0.475	2.104
4. Conscientiousness	−0.524 ^∗∗∗^	0.439 ^∗∗∗^	0.613 ^∗∗∗^	1					0.445	2.247
5. Agreeableness	−0.418 ^∗∗∗^	0.595 ^∗∗∗^	0.499 ^∗∗∗^	0.542 ^∗∗∗^	1				0.530	1.886
6. Ethnocultural empathy	−0.175 ^∗^	0.312 ^∗∗∗^	0.414 ^∗∗∗^	0.324 ^∗∗∗^	0.268 ^∗∗^	1			0.691	1.448
7. Self‐Esteem	−0.471 ^∗∗∗^	0.497 ^∗∗∗^	0.569 ^∗∗∗^	0.625 ^∗∗∗^	0.506 ^∗∗∗^	0.231 ^∗∗^	1		0.503	1.987
8. Cultural competence	−0.220 ^∗∗^	0.310 ^∗∗∗^	0.506 ^∗∗∗^	0.366 ^∗∗∗^	0.310 ^∗∗∗^	0.621 ^∗∗∗^	0.435 ^∗∗∗^	1		

^∗^ *p* < 0.05.

^∗∗^ *p* < 0.01.

^∗∗∗^ *p* < 0.001.

### 3.4. Effects of Independent Variables on Cultural Competence

While correlations of independent variables below 0.9 suggest the absence of multicollinearity, we further verified this by examining variance inflation factors (VIF). Multicollinearity was not a concern, as all variables showed a tolerance above 0.1 and a VIF below 10 (Table 4). Additionally, the Durbin–Watson statistic was 1.894, conforming the independence of residuals.

To analyze the variables affecting cultural competence, multiple regression analysis was conducted, and the results are as follows (Table 5). In Model 1, demographic and experiential variables (gender, age, clinical experience, and English proficiency) were entered. The model was statistically significant, *F* (4, 148) = 4.172, *p* < 0.01, explaining 10.1% of the variance in cultural competence (*R* ^2^ = 0.101). Among these variables, English proficiency was found to be a significant positive predictor of cultural competence (*β* = 0.249, *t* = 3.113, *p* < 0.01).

**Table 5:** Predictors of cultural competence: Multiple regression analysis (*N* = 153).

Variables	Model 1		Model 2	
	*β*	*t*	*β*	*t*
Gender (dummy)	0.090	1.017	−0.007	0.097
Age	0.113	0.458	−0.151	−0.797
Clinical experience (month)	0.069	0.286	0.235	1.273
English proficiency	0.249	3.113 ^∗∗^	0.179	2.794 ^∗∗^
Neuroticism			−0.002	−0.028
Extraversion			−0.157	−1.931
Openness			0.181	2.145 ^∗^
Conscientiousness			−0.074	−0.837
Agreeableness			0.038	0.469
Ethnocultural empathy			0.557	7.861 ^∗∗∗^
Self‐esteem			0.194	2.289 ^∗^
	*R* ^2^ = 0.101		*R* ^2^ = 0.528	
			*Δ* *R* ^2^ = 0.426	
	*F* = 4.172^∗∗^		*F* = 18.191^∗∗∗^	

^∗^ *p* < 0.05.

^∗∗^ *p* < 0.01.

^∗∗∗^ *p* < 0.001.

In Model 2, personality traits, ethnocultural empathy, and self‐esteem were added to the regression model. The overall model was significant, *F* (11, 141) = 18.191, *p* < 0.001, and accounted for 52.8% of the variance in cultural competence (*R* ^2^ = 0.528, *Δ* *R* ^2^ = 0.426). Among the predictors, openness (*β* = 0.181, *t* = 2.145, *p* < 0.05), ethnocultural empathy (*β* = 0.557, *t* = 7.861, *p* < 0.001), self‐esteem (*β* = 0.194, *t* = 2.289 *p* < 0.05), and English proficiency (*β* = 0.179, *t* = 2.794, *p* < 0.01) were identified as significant positive predictors of cultural competence. These findings indicate that occupational therapists with higher levels of English proficiency, openness, ethnocultural empathy, and self‐esteem tend to demonstrate stronger cultural competence.

## 4. Discussion

The world is rapidly evolving into a multicultural society, and South Korea is also transforming into a nation where diverse cultures coexist. Occupational therapists are healthcare professionals who provide medical services through interactions with clients from various cultural backgrounds [29]. Therefore, it is essential to identify the factors that influence the cultural competence required of occupational therapists in order to enhance their professional capabilities. This study aimed to examine the factors affecting the cultural competence of occupational therapists. The results revealed that English proficiency, openness, ethnocultural empathy, and self‐esteem significantly influenced their cultural competence.

First, among the general characteristics of the participants, English proficiency was found to influence cultural competence. Language ability is essential for understanding diverse cultures and facilitating effective communication [30]. In this global era, professionals across various fields continually strive to improve their English proficiency to enhance their competitiveness and gain exposure to diverse cultures and learning experiences [31]. English, the world’s common language, serves as a link for the exchange of ideas and the integration of cultures [32]. Previous studies have also reported that language learning promotes both multicultural and professional competence among healthcare professionals [15, 33, 34]. Taken together, high levels of English proficiency facilitate easier access to knowledge concerning diverse cultural groups and their respective worldviews. Occupational therapists who work with clients from diverse cultural backgrounds in community and clinical settings can enhance their multicultural competence by improving their English proficiency and engaging in active communication with other professionals.

Second, openness, one of the Big Five personality traits, was found to affect cultural competence. Individuals who are open to experience tend to hold positive attitudes towards multiculturalism, which in turn promotes higher cultural competence [35]. They are generally receptive to multicultural experiences and cultural diversity, which enables them to integrate different perspectives and generate creative solutions [36]. Furthermore, individuals with high level of openness are more open‐minded, accepting of people from different cultural backgrounds, and willing to engage with diversity [37]. Those who are open to diverse experiences and environments are more likely to embrace cultural diversity and adjust their perspectives and attitudes accordingly. Such experiences and attitudes may enhance multicultural competence, and the findings of this study, as well as those of previous research, support this relationship.

Third, the results of this study showed that multicultural empathy, along with openness, influenced the cultural competence of occupational therapists. Individuals with high levels of multicultural empathy are able to understand and accept others from different cultural backgrounds, which in turn promotes their multicultural competence [38]. Previous studies have suggested that multicultural empathy facilitates cultural awareness, cultural knowledge, and cultural skills [39, 40]. Furthermore, openness has been identified as an important personality trait that facilitates cultural intelligence and engagement in intercultural interactions [41]. Consistent with these findings, the present study indicates that individuals with greater multicultural empathy are more competent when interacting with people from diverse cultures. Multicultural empathy fosters openness to exploring and understanding cultural differences (such as values, beliefs, and norms) and enhances communication skills when engaging with individuals from various cultural backgrounds. Therefore, ethnocultural empathy contributes to facilitating communication in diverse situations, reducing cultural prejudice, and promoting effective and appropriate interactions in clinical practice through an open attitude toward different cultures. These skills represent core competencies for healthcare professionals, including occupational therapists.

Lastly, self‐esteem was also found to affect the cultural competence of occupational therapists. Self‐esteem refers to an individual’s evaluation of their own worth and reflects a positive or negative attitude toward the self [42]. According to Song and Yang, self‐esteem has a significant positive correlation with cultural competence, suggesting that a higher level of self‐esteem may be essential for the development of multicultural competence [8]. Self‐esteem, as a secure sense of self, is known to facilitate self‐reflection and openness [40]. Therefore, individuals with high self‐esteem are less likely to feel threatened when acknowledging their own biases and are more open to engaging with diverse cultures. Interacting with culturally different patients can create a sense of uncertainty; however, self‐esteem may serve as a psychological buffer against anxiety induced by uncertainty in multicultural healthcare settings [43]. Therefore, individuals with higher self‐esteem tend to tolerate for uncertainty and interact effectively with patients from different cultural background, and as a result, they can be culturally competent.

Multicultural competence is a key professional skill for occupational therapists, especially as the number of people from diverse cultural backgrounds continues to increase. This study suggests that openness to others, cultural empathy, and self‐esteem are essential for promoting multicultural competence. Campinha‐Bacote’s model of cultural competence conceptualizes cultural competence not merely as knowledge or skills, but as an ongoing developmental process [6]. From this perspective, the ethnocultural empathy identified in this study may facilitate cultural awareness and intercultural interactions, openness may promote cultural desire and the acquisition of cultural knowledge, and self‐esteem may enhance cultural skills and engagement in actual cultural encounters. Therefore, these factors can be understood as having complementary theoretical associations with components of Campinha‐Bacote’s cultural competence model. The American Occupational Therapy Association has emphasized the importance of regular and mandatory cultural competence training and education for healthcare professionals [4]. Therefore, organizations should provide ongoing cultural competence training to ensure that healthcare environments remain inclusive and responsive to the cultural needs of clients and their families. In addition, it is necessary to establish an educational foundation that strengthens the cultural competence of occupational therapy students through both academic instruction and clinical training experiences.

This study has several limitations. First, the participants were limited to occupational therapists in South Korea, and the relatively small sample size restricts the generalizability of the findings. Therefore, caution is required when interpreting the results. Second, as the study relied on self‐report questionnaires, the accuracy of the measurements may have been affected by subjective bias. To minimize this potential error, repeated and clear explanations of the questionnaire procedure are recommended in future research. Third, while multicultural empathy emerged as the most potent predictor of multicultural competence, it is important to note that these constructs share a significant conceptual foundation, as both involve navigating interactions with culturally diverse individuals. This inherent overlap suggests that they may be conceptually intertwined. Therefore, future research should aim to further clarify the distinct boundaries between these constructs to ensure theoretical precision. Furthermore, this study focused only on personality factors, ethnocultural empathy, and self‐esteem as variables influencing multicultural competence. Future studies should consider a broader range of individual, educational, and organizational factors to provide a more comprehensive understanding of multicultural competence among occupational therapists.

## 5. Conclusion

This study investigated the interrelationships among occupational therapists’ general characteristics, personality traits, ethnocultural empathy, self‐esteem, and cultural competence. The findings identified English proficiency, openness, ethnocultural empathy, and self‐esteem as significant predictors of cultural competence. These results underscore the importance of enhancing occupational therapists’ multicultural competence through targeted interventions. Specifically, the development of comprehensive language education initiatives, exposure to diverse cultural experiences, and the implementation of psychological support programs are recommended. Furthermore, integrating cultural competence training into both clinical practice and academic curricula is imperative to foster practitioners’ ability to deliver culturally responsive occupational therapy services.

## Author Contributions

Conceptualization: O.N.L. and G.A.P.; methodology: O.N.L. and G.A.P.; formal analysis: O.N.L. and G.A.P.; investigation: G.A.P.; writing and original draft preparation: O.N.L. and G.A.P.; funding acquisition: G.A.P.

## Funding

This study was supported by Chosun University, 10.13039/501100002457, K207830003, 2024.

## Ethics Statement

This study was approved by the Dongshin University Institutional Review Board (IRB No. 1040708‐202001‐SB‐002). All participants signed the written consent form prior to participating in the study.

## Conflicts of Interest

The authors declare no conflicts of interest.

## Data Availability

The data used in this study are available from the corresponding author upon request. The data are not publicly available due to privacy or ethical restrictions.
